# The Association Between Outdoor Ambient Temperature and Depression and Mania: An Ecological Momentary Assessment Study

**DOI:** 10.1192/bjo.2024.130

**Published:** 2024-08-01

**Authors:** Pip Clery, David Osborn, Joseph Hayes, Annie Jeffery, Jen Dykxhoorn

**Affiliations:** 1 University College London, London, United Kingdom; 2 Camden and Islington NHS Foundation Trust, London, United Kingdom

## Abstract

**Aims:**

Heat exposure can negatively impact mental health. Evidence for the effect of temperature on mood disorders is inconsistent. Current studies exploring the link between temperature and mood disorders are limited by poor temporal and geographical resolution. We aimed to use ecological momentary assessment (EMA) to investigate the effect of real-time temperature on depressive and manic symptoms. We hypothesised higher temperatures would be associated with increased depressive and manic symptoms.

**Methods:**

We used EMA data from the digital platform and smartphone app juli to investigate the effect of real-time mean and maximum ambient temperature on depressive and manic symptoms in adults with depression and bipolar disorder. Depressive and manic symptoms were assessed using the Patient Health Questionnaire-8 and the Altman Self Rating Mania score, respectively. Time- and location-specific temperature data were collected from participants’ smartphone geolocation on a 5-by-5 km resolution grid. We analysed data using negative binomial mixed-effects regression models, controlled for demographic and weather variables, and stratified by season.

**Results:**

We analysed data from 4,000 participants with depressive symptoms and 2,132 with manic symptoms, between July 2021 and March 2023. We found that each 1°C increase in mean daily temperature in the preceding two weeks was associated with a 0.2% reduction in depressive symptom scores (IRR 0.998, 95%CI 0.997–0.999). This association was most pronounced in the spring (IRR 0.995, 95%CI 0.992–0.999). For manic symptoms, we found that each 1°C increase in mean temperature in the preceding two weeks was associated with a 0.4% increase in manic symptom scores (IRR 1.004, 95%CI 1.001–1.007), with the strongest association observed in the autumn (IRR 1.011, 95%CI 1.002–1.020). Associations between maximum temperature and depressive and manic symptoms followed a similar pattern.

**Conclusion:**

We found evidence that higher temperatures were associated with decreased depressive symptoms and increased manic symptoms, indicating a complex relationship between temperature and mood disorder symptoms. With globally rising temperatures due to climate change, there is a need to understand the impact of heat on mental health symptoms to provide targeted support. This study demonstrates the potential for using novel data sources and EMA methods to inform our understanding of the link between climate and mental health, although there is a need for improved data collection to realise the potential of these methods. Clinically, our findings highlight opportunities for risk stratification and targeted interventions based on local temperature patterns.

